# Dr. Purves Stewart's Reply

**Published:** 1907-11-09

**Authors:** 


### HYPNOTISM IN ITS RELATION TO HYSTERIA AND TO PSYCHASTHENIA.

Dr. Purves Stewart's Reply.

Your correspondent Dr. Bryan seems to have misunderstood the references to hypnotism in its relation to hysteria and to psychasthenia which appeared under my "Notes on Current Neurology" of the 19th ult. Part of this misapprehension is due, perhaps, to the extremely condensed form in which I necessarily had to present the experiences of Professor Raymond, whose lectures on Neuroses and Psycho-neuroses appeared *in extenso* a short time ago in the French medical journals. It is recognised by all modern neurologists that one of the essential features of the syndrome known as hysteria is an abnormal susceptibility to suggestion, whether this be from without (hetero-suggestion) or from within (auto-suggestion). And in hysterical subjects the hypnotic sleep can be produced with special ease. In this sense hypnotism is an essential part of hysteria. This point has recently been elaborated in a brilliant lecture by M. Babinski (*Ma Conception de l'Hystérie, et de l'Hypnotisme*, 1906). Inasmuch as many of the primary phenomena of hysteria can be artificially produced or caused to disappear in suitable subjects by means of hypnotic suggestion, hypnotism is a remedy which, in selected cases, we are sometimes justified in recommending. But we must remember that even in hysteria hypnotism is not a panacea and should not be employed indiscriminately.

Psychasthenia, on the other hand, as Janet has shown, is a disease essentially different from hysteria, though in practice the two affections are not infrequently combined. The psychasthenic, like the poet, is born, not made; he possesses certain stigmata which are an integral part of his fundamentally degenerate nervous constitution, stigmata which no degree of hypnotic suggestion can eradicate. And if the psychasthenic does not already happen to suffer from hysteria, hypnotism may do positive harm by superadding an artificial state of hysteria. Such is the experience of Raymond and of the Salpetrière school, with which I am in entire agreement. In the treatment of psychasthenia we endeavour to modify the mental, moral, and physical environment; we encourage the patient to overcome his various symptoms, but we refrain from throwing him into the hypnotic sleep. Hypnotism and psychotherapeutics are not synonymous terms. True, they both depend on suggestion; but the difference in degree is so great, and the clinical phenomena aimed at so different, that the practical physician has no doubt in his mind as to what he means by the two expressions.

To discuss fully the subjects of hysteria and of psychasthenia would demand more space than the columns of The Hospital can afford, but the essence of the particular point under discussion seems to me to be that whilst in hysteria we may, in suitable cases, profoundly alter the symptoms by means of hypnotism, in psychasthenia we cannot hope to abolish the patient's fundamentally degenerate nervous constitution, and therefore in cases of pure psychasthenia hypnotism is worse than useless.

